# Integrated application of multi-omics provides insights into cold stress responses in pufferfish *Takifugu fasciatus*

**DOI:** 10.1186/s12864-019-5915-7

**Published:** 2019-07-08

**Authors:** Xin Wen, Yadong Hu, Xinyu Zhang, Xiaozhen Wei, Tao Wang, Shaowu Yin

**Affiliations:** 1 College of Life Sciences, College of Marine Science and Engineering, Nanjing Normal University, Nanjing, 210023 China; 2 Co-Innovation Center for Marine Bio-Industry Technology of Jiangsu Province, Lianyungang, 222005 Jiangsu China

**Keywords:** Environmental stress, Multi-omics, Low temperature, mRNA-protein-metabolite, *Takifugu fasciatus*

## Abstract

**Background:**

*T. fasciatus* (*Takifugu fasciatus*) faces the same problem as most warm water fish: the water temperature falls far below the optimal growth temperature in winter, causing a massive death of *T. fasciatus* and large economic losses. Understanding of the cold-tolerance mechanisms of this species is still limited. Integrated application of multi-omics research can provide a wealth of information to help us improve our understanding of low-temperature tolerance in fish.

**Results:**

To gain a comprehensive and unbiased molecular understanding of cold-tolerance in *T. fasciatus*, we characterized mRNA-seq and metabolomics of *T. fasciatus* livers using Illumina HiSeq 2500 and UHPLC-Q-TOF MS. We identified 2544 up-regulated and 2622 down-regulated genes in the liver of *T. fasciatus*. A total of 40 differential metabolites were identified, including 9 down-regulated and 31 up-regulated metabolites. In combination with previous studies on proteomics, we have established an mRNA-protein-metabolite interaction network. There are 17 DEMs (differentially-expressed metabolites) and 14 DEGs-DEPs (differentially co-expressed genes and proteins) in the interaction network that are mainly involved in fatty acids metabolism, membrane transport, signal transduction, and DNA damage and defense. We then validated a number of genes in the interaction network by qRT-PCR. Additionally, a number of SNPs (single nucleotide polymorphisms) were revealed through the transcriptome data. These results provide key information for further understanding of the molecular mechanisms of *T. fasciatus* under cold stress.

**Conclusion:**

The data generated by integrated application of multi-omics can facilitate our understanding of the molecular mechanisms of fish response to low temperature stress. We have not only identified potential genes and SNPs involved in cold tolerance, but also show that some nutrient metabolites may be added to the diet to help the overwintering of *T. fasciatus*.

**Electronic supplementary material:**

The online version of this article (10.1186/s12864-019-5915-7) contains supplementary material, which is available to authorized users.

## Background

Temperature has profound effects on the physiology and behavior of ectotherms, especially teleosts [1]. Within the appropriate temperature range, fish have the capacity to cope with natural temperature changes, such as daily and seasonal changes [2]. However, low temperature can cause irreversible damage [3], especially in farmed species that are unable to escape unfavorable environmental conditions. Low temperature causes alterations in the cellular biological functions, structures, and metabolism, as well as folding, assembly, activity, and stability of proteins [4, 5]. However, we have only poor knowledge about the mechanisms underlying these organisms’ responses to low temperature. Investigating how and to what extent organisms adapt to low temperature at various molecular and physiological levels could provide a better understanding of the molecular mechanisms underlying environmental adaptation.

It has been shown that fish can gradually establish cold adaptive phenotypes through extensive biochemical, metabolic and physiological regulations [6]. Well-defined biochemical and physiological acclimations include producing temperature-specific isozymes [7], altering the content of membrane lipid and the degree of fatty acid unsaturation [8], recruiting different muscle fiber types [9], synthesizing molecular chaperones [10], and altering mitochondrial densities and their properties [11]. However, this amount of information is very limited for a comprehensive understanding of the changes in the organisms. Recently, a rise of omics applications has made it convenient to acquire bioinformation in many studies, especially the screening of key pathways or major genes for the regulation of economically-important traits. Transcriptomics has already been employed to assess responses to cold environment [12, 13]. A comparison of the zebrafish and tilapia transcriptomes revealed that the FoxO signal regulates the low-temperature limit [14]. The comparative studies of transcriptomes and microRNAomes showed that notothenioid lineages were adapted to cold by evolutionary modulation of the multi-functional TGF-β signaling pathway [15]. Metabolomics has also been applied to study the metabolic differences of gilthead sea bream under low-temperature stress, and that study found that the most perturbed metabolic pathways are related to methionine cycle in liver at low water temperature (11 °C) [16]. In the past decade several studies have elaborated on the mechanisms involved in the responses of fish to low temperature. However, the molecular mechanisms of teleost’s response to low-temperature stress have not yet been analyzed using multi-omics.

The biological phenomena are complex and variable, and the regulation of gene expression is complicated. When conducting a single-omics study, the conclusions are often not comprehensive. Therefore, there is a bottleneck in the study of single omics. Multi-omics combines two or more -omics research methods, such as genomics, transcriptomics, proteomics, and/or metabolomics. In teleosts, the combination of transcriptomics and proteomics has revealed some important biological phenomena. For example, it has highlighted the molecular mechanism of dopamine in reproduction of goldfish [17]; revealed that tilapia gut and liver may collaborate immunologically [18]; revealed that dietary vegetable oil can cause indelible injuries to intestines of Atlantic salmon (*Salmo salar*) [19]; and explored the differential genes and proteins in the red skeletal muscle of rainbow trout (*Oncorhynchus mykiss*) and the gilthead seabream (*Sparus aurata*) during sustained swimming [20]. In addition, the combination of proteomics and metabolomics can be used to discriminate fish liver tumors in wild flatfish [21]. Transcriptomics and metabolomics have also been used to identify health impacts of exposure to copper in fish [22]. Unfortunately, the use of multi-omics to study the response mechanisms of fish under low-temperature stress is still close to negligible, which greatly hinders our comprehensive understanding of this biological phenomenon.

As an anadromous and widely distributed species in the South China Sea, the East China Sea, and inland waters in China and Korean Peninsula [23], *Takifugu fasciatus* (*T. fasciatus*) (pufferfish) is an important farmed fish of high commercial value [24]. The consumption of *T. fasciatus* has a long history in the middle and lower reaches of the Yangtze River in China [25]. Furthermore, the pufferfish is also popular in Korea [26] and Japan [27]: pufferfish sashimi is widely known. As a kind of warm-water fish [28], *T. fasciatus* has an optimum growing temperature between 23 °C and 32 °C [29]. However, in farms as well as rivers, the water temperature in winter is far below that optimal growth temperature, which causes massive deaths of *T. fasciatus* and leads to large economic losses. Therefore, it is necessary to understand the mechanisms of low-temperature tolerance in *T. fasciatus*.

In the present study, transcriptomics and metabolomics were used to conduct comparative quantitative omics analysis of *T. fasciatus* liver after exposure to low-temperature water, and the results were combined with our previous proteomics research to further broaden our perspective on the mechanisms of *T. fasciatus* responding to low-temperature stress. The results not only provided new and important information about the cold response of *T. fasciatus*, but also illustrated more completely the relationship between warm-water fish and low-temperature environments.

## Results

### Transcriptome analysis of *T. fasciatus* liver under cold stress

In order to identify mRNA expression profiles in *T. fasciatus* liver under cold stress six cDNA libraries representing the CG (control groups: G1, G2, G3) and EG (experimental group: Ga, Gb, Gc) were constructed with total RNA and subjected to Illumina deep sequencing. In total, 58,246,984, 62,328,458, and 49,318,414 raw reads were obtained from the CG libraries G1, G2, and G3, respectively, and 71,189,264, 51,105,490, and 61,379,788 raw reads were obtained from the libraries Ga, Gb, and Gc, respectively. After quality filtering, approximately 96.46, 97.07, and 96.56% clean reads from CG and 97.01, 96.13, 97.15% clean reads from EG libraries qualified for assembling (Additional file 1: Table S1). These clean reads were mapped to the genome of *T. fasciatus*; approximately 89.94% (G1), 89.54% (G2), and 89.42% (G3) clean reads from CG and 90.03% (Ga), 90.34% (Gb), 89.91% (Gc) clean reads from EG libraries were located (Additional file 2: Table S2). In all, we identified 20,991 genes, of which 19,924 were able to be matched and annotated by the genome (Additional file 3: Table S3). Subsequently, 2544 up-regulated and 2622 down-regulated genes (differentially expressed genes, DEGs) were identified in *T. fasciatus* liver, which showed significantly different expression between CG and EG (Additional file 4: Figure S1).

A total of 273 GO clusters that changed significantly (*p*-value < 0.05) after low temperature stress were identified (Additional file 5: Table S4). The gene ontology (GO) analysis of DEGs produced three major functional categories: biological process, cellular component, and molecular function. Among the molecular function category, “binding” was most commonly represented, followed by “ion binding”. Gene involved in the “intracellular” and “intracellular part” were notably represented in the cellular components. In the category of biological process, a significant proportion of clusters were assigned to “cellular response to DNA damage stimulus” and “macromolecule catabolic process” (Additional file 6: Figure S2). By performing KEGG (Kyoto Encyclopedia of Genes and Genomes) pathway enrichment analyses, a total of 12 pathways (Additional file 7: Table S5) that changed significantly (*p* -value < 0.05) after the low-temperature treatment were identified. Among these pathways, ribosome biogenesis in eukaryotes, glyoxylate and dicarboxylate metabolism, RNA transport, PPAR signaling pathway, and fatty acid biosynthesis, among others, were significantly enriched.

### Metabolome analysis of *T. fasciatus* liver under cold stress

**Fig. 1:**
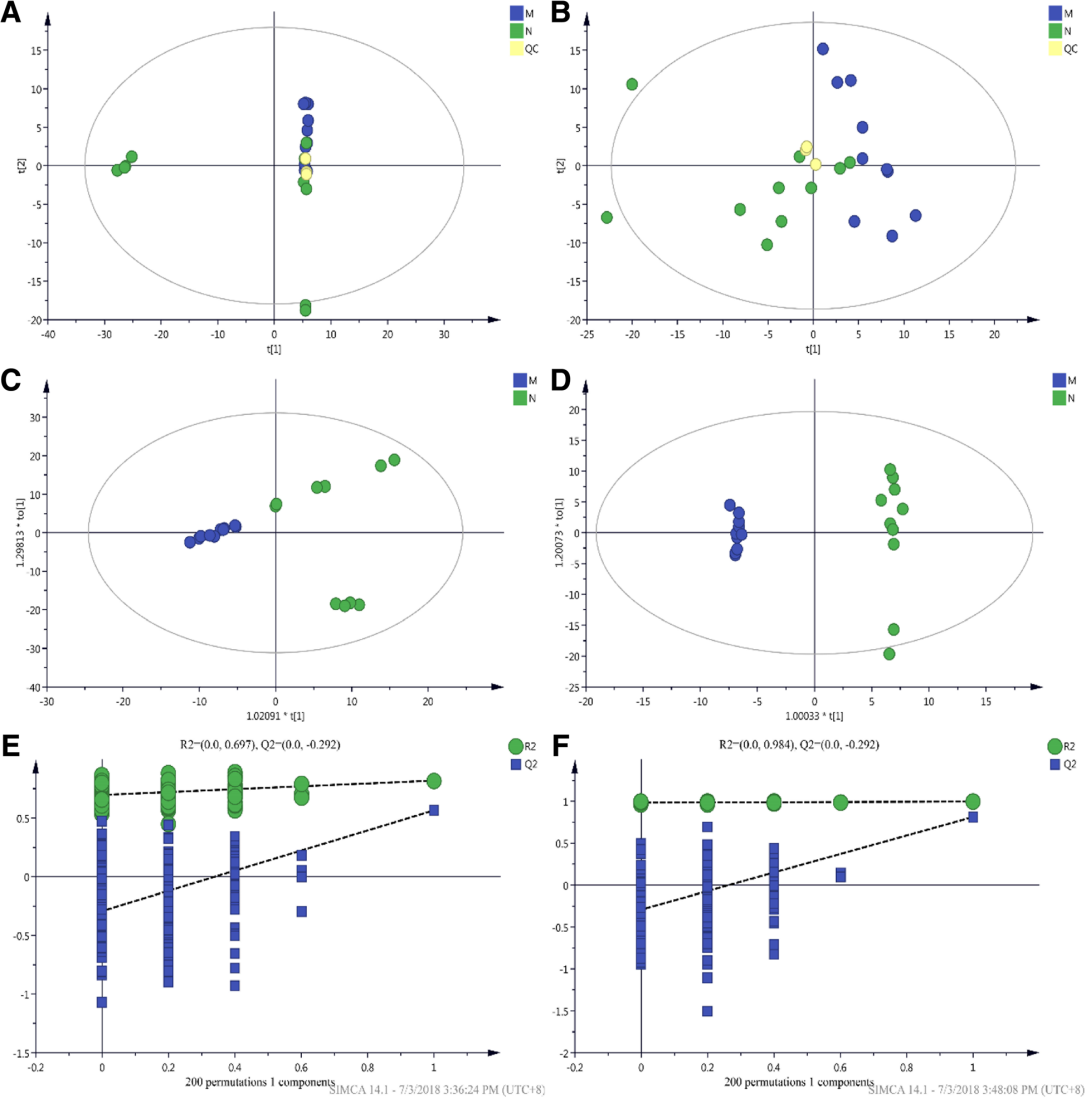
Quality analysis of metabolomics data. **a** PCA (Principal Component Analysis) score diagram of samples in positive ion mode. **b** PCA score diagram of samples in negative ion mode. **c** OPLS-DA (orthogonal partial least-squares-discriminant analysis) score diagram for positive ion mode. **d** OPLS-DA score diagram for negative ion mode. **e** OPLS-DA permutation test for positive ion mode. **f** OPLS-DA permutation test for negative ion mode

To understand the metabolome changes associated with *T. fasciatus* under cold stress, an untargeted metabolomics analysis was performed in livers using a UHPLC-Q-TOF MS platform. A total of 9464 metabolic ion peaks were extracted, including 4085 positive ion peaks and 5379 negative ion peaks. In the positive ion mode, a total of 40 differential metabolites were identified (Table 1), including 9 down-regulated and 31 up-regulated metabolites. The PCA (Principal Component Analysis) model obtained by 7-fold cross-validation showed that the QC (Fig. 1a, b) samples in the positive and negative ion modes were closely clustered, indicating that the experiment was reproducible. The OPLS-DA (orthogonal partial least-squares-discriminant analysis) model of the example set was established (Fig. 1c, d); the model evaluation parameters (Positive ion mode: R2Y = 0.82 cum, Q2 = 0.566 cum; Negative ion mode: R2Y = 0.997 cum, Q2 = 0.812 cum) obtained by 7-fold cross-validation indicated that the model is stable and reliable. The permutation test established 200 OPLS-DA models by randomly changing the order of the categorical variables Y to obtain the R2 and Q2 values of the stochastic model (Fig. 1e, f). All Q2 points from left to right are lower than the original blue Q2 points on the right side, indicating that the model is robust and reliable without overfitting. In summary, the instrument analysis system and the test data of this experiment were stable and reliable.

**Table 1:** Differential metabolites in positive ion mode

Name	Description	VIP	Fold Change	*P*-value
M853T235_1	1,2-dioleoyl-sn-glycero-3-phosphatidylcholine	2.59884	1.588503	2.61E-05
M245 T288	Uridine	1.40599	0.632063	7.1E-05
M136T282_2	Adenine	9.63034	1.831853	0.001092
M383 T74	25-hydroxyvitamin D3	1.41132	2.041806	0.002168
M667 T935	Stachyose	2.21459	1.180736	0.002686
M517 T250	Taurodeoxycholic acid	1.13317	2.087169	0.002864
M258 T734	Glycerophosphocholine	2.70521	0.663651	0.003081
M336 T97	Isopentenyladenosine	1.33553	1.409782	0.004483
M801 T242	PC(16:0/16:0)	1.39795	1.387887	0.004601
M498 T345	Taurocholate	3.73214	2.041481	0.006176
M324 T809	3′-O-methylcytidine	1.99467	1.266727	0.006812
M137T299_3	Hypoxanthine	8.84071	1.155196	0.006871
M198 T556	D-mannose	1.00698	1.906164	0.007194
M220 T487	Pantothenate	1.91888	1.38217	0.007796
M827 T238	PC(20:5(5Z,8Z,11Z,14Z,17Z)/20:5(5Z,8Z,11Z,14Z,17Z))	1.66436	1.261062	0.01511
M102 T610	Betaine aldehyde	1.54531	1.255756	0.016507
M803 T242	Thioetheramide-PC	2.03214	1.243288	0.018703
M325 T763	Isomaltose	2.07078	1.698419	0.019203
M204 T554	Acetylcarnitine	8.19603	1.160316	0.020809
M152T473_2	2-hydroxyadenine	1.2236	0.837138	0.02148
M118T491_2	Betaine	2.23583	0.742234	0.021849
M311 T59	(4Z,7Z,10Z,13Z,16Z,19Z)-4,7,10,13,1 6,19-docosahexaenoic acid	1.45373	1.429623	0.022959
M284 T473	Guanosine	1.60775	0.846529	0.028553
M162T653_2	L-carnitine	8.28178	1.129482	0.035901
M348 T755	Adenosine 3′-monophosphate	2.57573	1.14958	0.036439
M496 T315	1-palmitoyl-sn-glycero-3-phosphocholine	1.91884	0.75864	0.037082
M130 T990	D-pipecolinic acid	1.05115	1.161867	0.043145
M268 T305	Adenosine	1.95144	1.189739	0.043667
M360 T721	Cellobiose	12.6562	1.459678	0.049378
M147 T990	L-lysine	1.20587	1.139057	0.059546
M325 T843	D-maltose	3.20298	1.232419	0.07064
M132 T470	L-leucine	1.03566	1.158161	0.071885
M613 T924	Glutathione disulfide	3.11101	1.156391	0.083255
M327 T79	Arachidonic acid (peroxide free)	2.44396	0.711068	0.083971
M522 T865	Maltotriose	3.08089	1.200343	0.087121
M298 T165	S-Methyl-5′-thioadenosine	2.67371	0.708227	0.087539
M829 T961	Maltopentose	1.71797	1.154751	0.090842
M123 T100	Nicotinamide	13.2201	1.283029	0.093743
M293 T783	EDTA	1.82258	0.451273	0.095864
M277 T845	gamma-L-glutamyl-L-glutamic acid	1.54325	1.202509	0.098591

### Multi-omics identification of key genes and metabolites

**Fig. 2:**
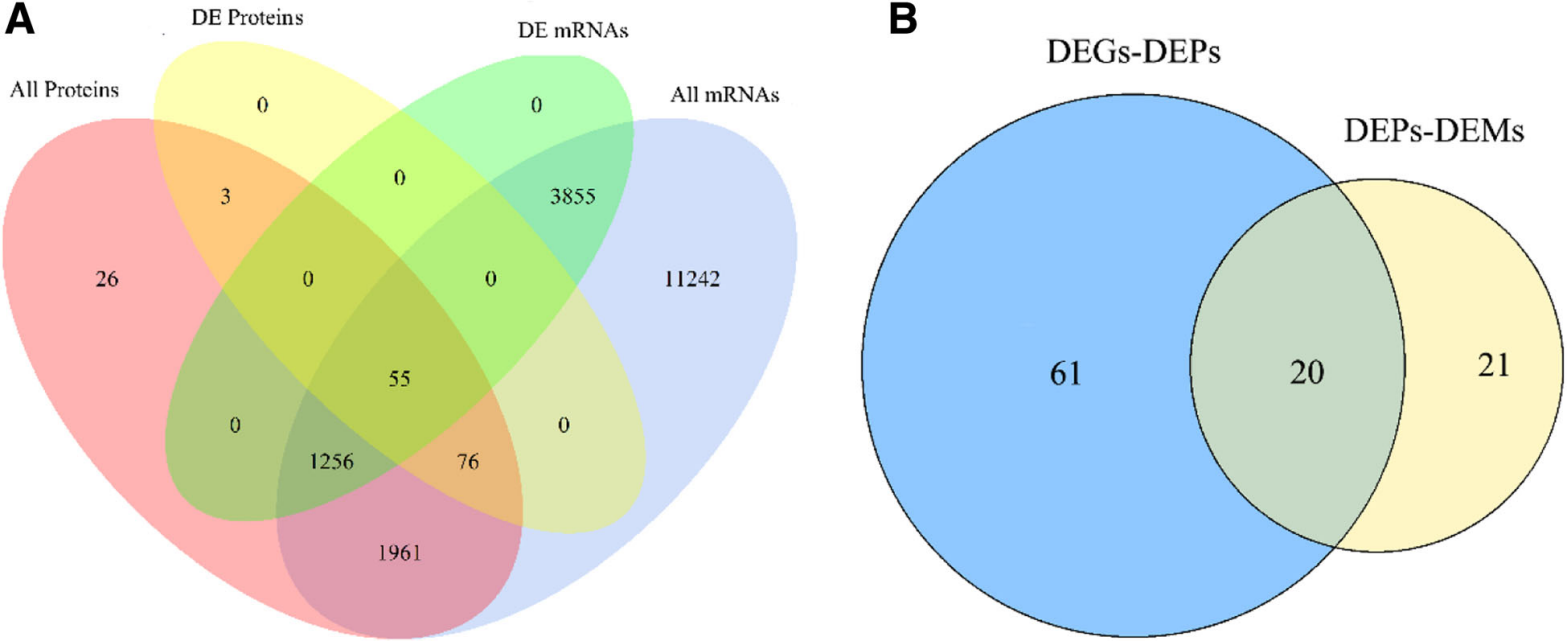
Venn diagrams describing the numbers of mRNAs, proteins and pathways that varied between EG (experimental group) and CG (control group). **a** the number of transcriptome and proteome at the quantitative and differential expression levels. **b** the number of pathways involved in DEGs-DEPs (differentially co-expressed genes and proteins) or DEPs-DEMs (associated DEPs and DEMs) and their commonalities

In this study, the transcriptome and proteome of *T. fasciatus* liver under cold stress were integrated by investigating whether changes in the protein levels correlated with changes in the corresponding transcripts. Based on the level of mRNA and protein expression, we classified the genes and proteins involved into five different types (DEGs-DEPs-SameTrend, DEGs-DEPs-Opposite, DEGs-NDEPs, NDEGs-DEPs, NDEPs-NDEGs, Fig. 2a; Additional file 8: Table S6), and the subsequent analysis was based on this classification. A total of 36 DEPs (differentially expressed proteins) were matched with corresponding DEGs, which included 18 up-regulated DEGs-DEPs and 18 down-regulated DEGs-DEPs that were well matched with the tendency of change in abundance of DEGs. On the other hand, 19 DEPs had opposite trends to the DEGs (Additional file 8: Table S6). Investigating the 55 co-expressed genes (DEGs-DEPs) revealed that they were involved in 81 metabolic pathways. Subsequently, combined proteome and metabolome analyses revealed that a total of 41 pathways were involved in DEPs-DEMs (associated DEPs and DEMs).

Genes produce proteins through complex transcription and translation processes to regulate the metabolism of organisms. Therefore, to obtain an overview of the correlation between the level of transcription, protein and metabolism, the correlation of 81 pathways (related to DEGs-DEPs) and 41 pathways (related to DEPs-DEMs) was analyzed. A total of 20 identical pathways (Fig. 2b) are involved in DEGs, DEPs and DEMs (Table 2). These pathways contained metabolic processes (e.g. pyrimidine metabolism; biosynthesis of unsaturated fatty acids; amino sugar and nucleotide sugar metabolism; valine, leucine and isoleucine degradation; glutathione metabolism; lysosomal degradation), membrane transport (e.g. ABC transporters), signal transduction (e.g. cAMP signaling pathway), organism systems (e.g. bile secretion; vitamin digestion and absorption; retrograde endocannabinoid signaling; serotonergic synapse; regulation of lipolysis in adipocytes; aldosterone synthesis and secretion; longevity regulating pathway) and diseases (e.g. central carbon metabolism in cancers; morphine addiction; alcoholism; Parkinson’s disease; insulin resistance).

**Table 2:** DEGs, DEPs and DEMs involved in the pathways

Pathway	Gene/Protein	Metabolite
Pyrimidine metabolism	Uridine phosphorylase 2^↑^	Uridine ^↓^
Biosynthesis of unsaturated fatty acids	Acyl-CoA desaturase^↑^	Arachidonic acid (peroxide free) ^↓^; (4Z,7Z,10Z,13Z,16Z,19Z)-4,7,10,13,16,19-Docosahexaenoic acid^↑^
Amino sugar and nucleotide sugar metabolism	N-acetyl-D-glucosamine kinase ^↓^	D-mannose^↑^
Valine, leucine and isoleucine degradation	Acetoacetyl-CoA synthetase^#^	L-leucine^↑^
Glutathione metabolism	Glutathione S-transferase omega-1^↑^	Glutathione disulfide ^↑^
Lysosome	Acid phosphatase ^↓^	D-mannose^↑^
ABC transporters	Bile salt export pump^↑^; Canalicular multispecific organic anion transporter 1^+^	Maltotriose^↑^;L-lysine^↑^; L-leucine^↑^;D-mannose^↑^; D-maltose^↑^;Betaine^↓^;
cAMP signaling pathway	Hormone-sensitive lipase^+^	Adenosine^↑^
Vitamin digestion and absorption	Retinol-binding protein 2^↑^	Pantothenate^↑^; Nicotinamide^↑^;
Bile secretion	Solute carrier family 2, facilitated glucose transporter member 1^↑^; Ubiquitin-associated protein 1-like^↑^; Bile salt export pump^↑^; Canalicular multispecific organic anion transporter 1+	Taurocholate^↑^; L-carnitine^↑^
Retrograde endocannabinoid signaling	Guanine nucleotide-binding protein subunit beta-4^↓^	Arachidonic acid (peroxide free) ^↓^; PC(16:0/16:0) ^↑^
Serotonergic synapse	Guanine nucleotide-binding protein subunit beta-4^↓^	Arachidonic acid (peroxide free) ^↓^;
Regulation of lipolysis in adipocyte	Hormone-sensitive lipase^+^	Arachidonic acid (peroxide free) ^↓^; Adenosine^↑^
Aldosterone synthesis and secretion	Hormone-sensitive lipase^+^	Arachidonic acid (peroxide free) ^↓^;
Longevity regulating pathway – worm	Acyl-CoA desaturase^↑^	Nicotinamide^↑^
Central carbon metabolism in cancer	Solute carrier family 2, facilitated glucose transporter member 1^↑^	L-leucine^↑^
Morphine addiction	Guanine nucleotide-binding protein subunit beta-4^↓^	Adenosine^↑^
Alcoholism	Guanine nucleotide-binding protein subunit beta-4^↓^	Adenosine^↑^
Parkinson disease	ATP synthase-coupling factor 6^↑^	Adenosine^↑^
Insulin resistance	Solute carrier family 2, facilitated glucose transporter member 1^↑^	Acetylcarnitine^↑^

“↑” superscripts indicate significantly up-regulated

“↓” superscripts indicate significantly down-regulated

“+” superscript indicates that the gene was significantly up-regulated at the mRNA level and significantly down-regulated at the protein level

“#” superscript indicates that the gene was significantly down-regulated at the mRNA level and significantly up-regulated at the protein level

**Fig. 3:**
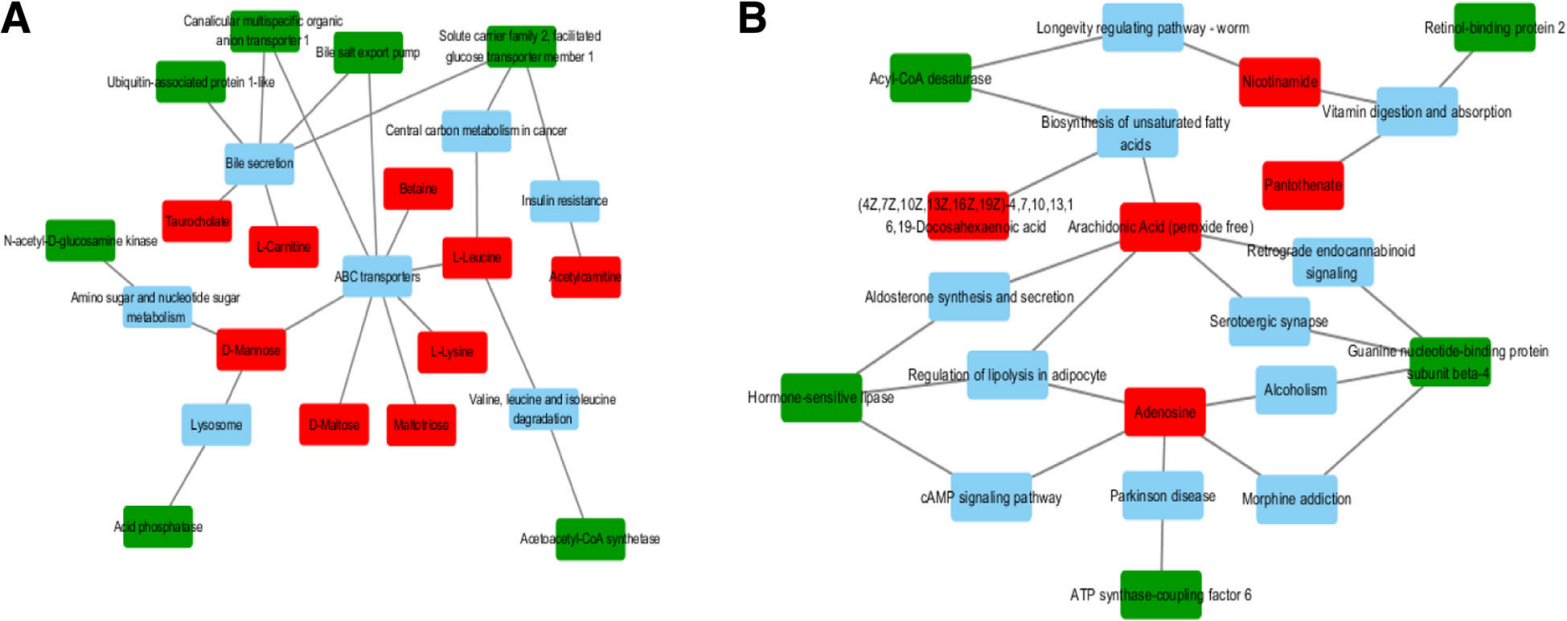
Interaction networks of the significant differentially co-expressed genes and metabolites analyzed by Cytoscape software (version 3.0.1). **a** Interaction networks related to the transmembrane transport of bile salts. **b** Interaction networks related to unsaturated fatty acids, vitamins and adenosine. Red indicates metabolite; green indicates co-expressed gene; blue indicates pathway

There were 17 DEMs (14 up-regulated with fold change > 1, and 3 down-regulated with fold change < 1) and 14 DEGs-DEPs (8 up-regulated, 3 down-regulated, 3 opposite) in these 20 pathways (Table 2). The interaction between them was clustered mainly into two clusters. One of these was related mainly to the transmembrane transport of bile salts (Fig. 3a), and the other to unsaturated fatty acids, vitamins and adenosine (Fig. 3b).

### Validation of selected genes by qPCR

We performed real-time qPCR analysis on some selected genes to provide additional mRNA transcript levels to validate our RNAseq results. The results of qPCR analysis revealed that most of these genes shared similar expression tendencies (log2fold change; EG vs CG) with those from transcriptomic data (Additional file 9: Table S7). Although some quantitative differences did exist between the two analytical platforms, the common characteristics of the proteomic data and the qRT-PCR analysis supported the reproducibility and reliability of the transcriptomic data.

### SNP detection and validation

For extended application of the RNA-Seq data, structural variations were discovered using the assembled transcriptome. Information on all SNPs detected was presented in Additional file 10: Table S8. A total of 23 potential SNPs were identified by detection in 5 co-expressed genes: 3 up-regulated and 2 down-regulated genes (Additional file 11: Table S9). Twenty-three SNPs were tested for experimental validation in fifty *T. fasciatus*. Among the 23 primer pairs designed for SNP validation, 19 could amplify target sequences. Within these amplified sequences, 13 SNPs were validated, suggesting that approximately 50% of the predicted SNPs were indeed true SNPs. The primers of validated SNPs are listed in Additional file 12: Table S10.

## Discussion

**Fig. 4:**
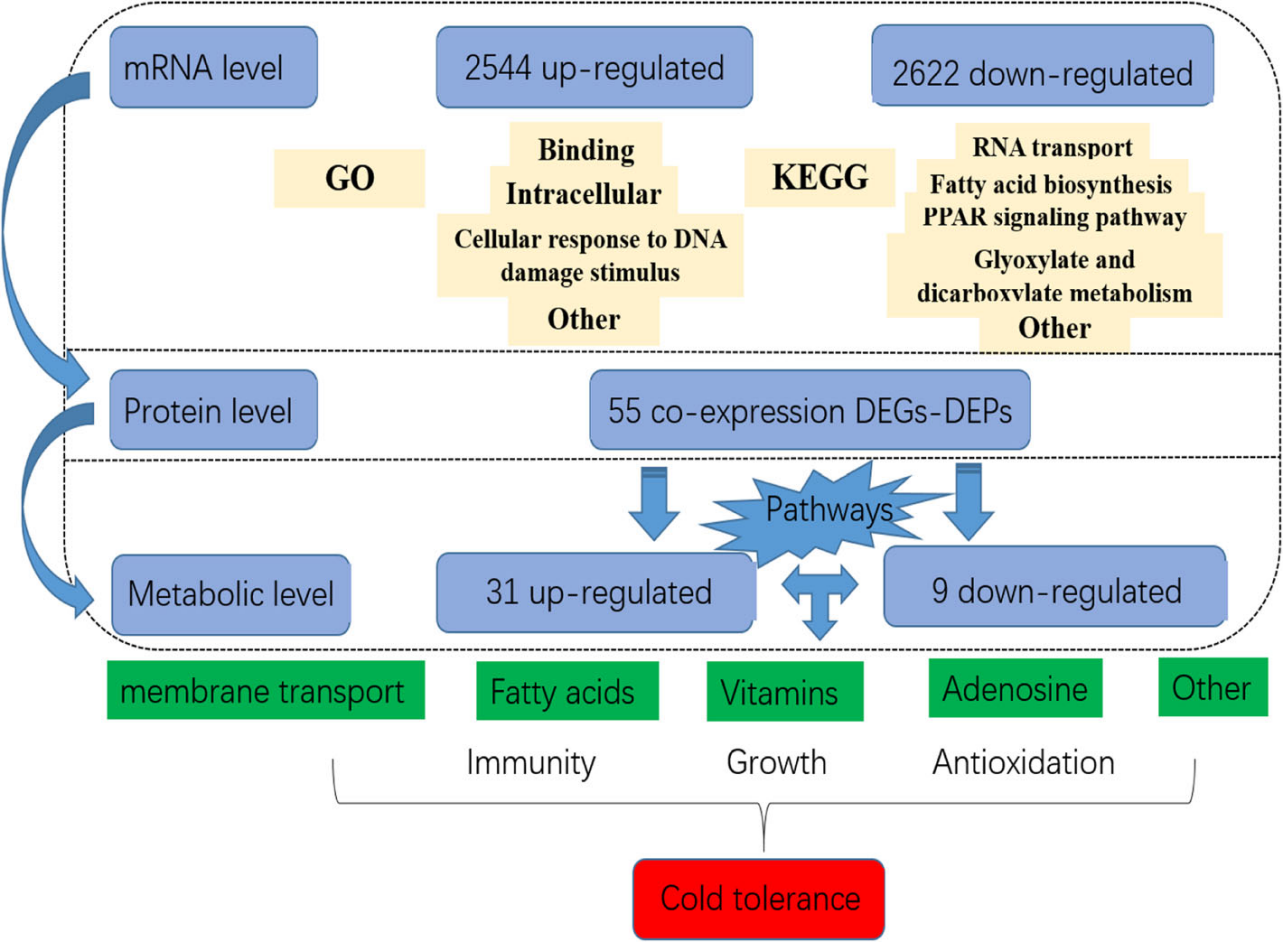
Model of possible molecular mechanisms of *T. fasciatus* under cold stress

In the present study, low temperature stress was associated with numerous co-expressed DEGs-DEPs and DEMs mainly involved in fatty acid metabolism, oxidative stress, immune system, membrane transport and signal transduction of *T. fasciatus* (Fig. 3; Table 2). These systems strongly responded to low-temperature stress, and the further exploration of cold stress may deepen our understanding of the molecular mechanisms in fish adaptation (Fig. 4).

### Fatty acids metabolism

The physical properties of phospholipid membranes are heavily dependent upon the saturation of their constituent fatty acids [30]. Maintaining an appropriate balance between saturated and unsaturated fatty acids, under a variable dietary supply, is therefore an essential compositional requirement for all living organisms. Both arachidonic acid (ARA) and (4Z,7Z,10Z,13Z,16Z,19Z)-4,7,10,13,16,19-docosahexaenoic acid (DHA) are important components of membrane phospholipids [31]. The enrichment of ARA and DHA is mainly related to Acyl-CoA desaturase (ACAD) [31], an enzyme involved in maintaining the balance between saturation and unsaturation of fatty acids. Under cold stress, ACAD was up-regulated in the liver of *T. fasciatus*, indicating that the production of unsaturated fatty acids was strengthened. This is consistent with the findings on carp responses to low-temperature stress [32].

In our metabolomics study, two types of unsaturated fatty acids showed opposite trends (ARA-down, DHA-up), perhaps because there is an antagonistic relationship between ARA and DHA [33, 34]. This has been reported in yellowtail liver, where ARA was incorporated into phosphatidylinositol more effectively than DHA, and the latter inhibited incorporation of ARA into phosphatidylcholine but not phosphatidylinositol [35]. Of course, a more credible reason was that in the combined multi-omics analysis, the metabolism of ARA was affected by other genes and pathways. Compared to the uncertainty around ARA, the up-regulation of DHA was undoubtedly the response of fish enhancing their tolerance of low-temperature environmental stress [36, 37]. In the endocrine system, ARA is regulated by hormone-sensitive lipase (HSL) through two pathways (Fig. 3b). HSL is a key enzyme in the mobilization of fatty acids from intracellular stores [38]. The findings on grass carp [39] and gilthead sea-bream [40] confirmed that dietary ARA can significantly stimulate HSL expression. On the other hand, in the nervous system, much evidence has accumulated indicating that the guanine nucleotide-binding protein subunit beta-4 (G protein) has a positive effect on the metabolism of ARA [41]. Therefore, down-regulation of this G protein would undoubtedly affect the metabolism of ARA. These results indicate that the metabolism of ARA in *T. fasciatus* subjected to low-temperature stress is regulated by various pathways and genes.

In terms of fatty acid synthesis, Acetoacetyl-CoA synthetase (AACS) is a ketone body-utilizing enzyme responsible for the synthesis of cholesterol and fatty acids from ketone bodies in lipogenic tissues such as liver and adipocytes [42]. In the present study, expression of AACS was down-regulated at the mRNA level, but was up-regulated at the protein level. Complex post-transcriptional mechanisms can be responsible for the inconsistency between mRNA and protein synthesis [43]. Previous research found that leucine supplementation was effective in increasing adiponectin concentration and in reducing total cholesterol concentration in rats [44]. Therefore, the up-regulation of AACS and L-leucine in *T. fasciatus* liver was associated with regulation of the synthesis of cholesterol and fatty acids in response to low-temperature stress.

### Membrane transport and signal transduction

Several lines of evidence clearly indicate that ABC transporters play an important role in the antioxidant mechanism of fish [45]. The bile salt export pump (BSEP) is an important member of the ABC transporter pathway. It is a membrane glycoprotein located on the hepatocyte membrane that mediates ATP-dependent transport of bile salt from the cells to the capillary bile duct [46]. Up-regulation of BSEP can enhance the transport of bile salts to prevent the production of oxygen free radicals and to decrease lipid peroxides and mitochondrial respiratory chain disorders, thereby protecting the function of biofilm and strengthening the immunity of organisms [47]. The blockage of bile salt transport by biliary obstruction induced a variety of liver diseases in humans and mice [48]. Therefore, the antioxidant role of BSEP in fish may be consistent with that in humans and mice.

In the ABC transporter pathway several organic anions are excreted into the bile via a canalicular multispecific organic anion transporter (cMOAT) [49]. The regulation of cMOAT expression is closely related to the liver function of an organism. Compared with the control group, synthesis of cMOAT decreased when the mRNA was unchanged or up-regulated, suggesting a post-transcriptional regulation mechanism. Further analysis found that the metabolic differences caused by low-temperature stress were directly related to the expression of cMOAT and BSEP. Our results show that up-regulation of BSEP was involved in the transport of oligosaccharides (D-Maltose and maltotriose), amino acids (L-lysine and L-leucine) and monosaccharides (D-mannose), whereas up-regulation of cMOAT was involved in the transport of mineral and organic ion (betaine) [50]. Among these up-regulated metabolites, D-mannose can enhance the organism immunity [51]; D-maltose and maltotriose can enhance energy metabolism; and L-leucine is mainly involved in the synthesis of cholesterol and fatty acids. The downregulation of betaine negatively affects fish growth, antioxidant defense and fatty acid synthesis [52]. These differential metabolites reflect the complex transport compensation mechanism of *T. fasciatus* in the response to low-temperature stress. Not only that, cMOAT and BSEP are related to the metabolism of taurocholate in the bile secretion pathways. Taurocholate supplementation has a potentiating effect on turbot (*Scophthalmus maximus*) [53], but has a negative impact on the growth of rainbow trout (*Oncorhynchus mykiss*) [54]. Therefore, it remains uncertain whether taurocholate up-regulation has any effect on the growth performance of *T. fasciatus* under low-temperature stress. However, taurocholate might play an important role in teleost fish in response to environmental temperature changes because rainbow trout - a cold-water fish - enhanced the metabolism of taurocholate under heat stress [55], whereas *T. fasciatus* - a warm-water fish - also strengthened the metabolism of taurocholate under cold stress. These results reflect the membrane transport mechanisms in response of *T. fasciatus* to low-temperature stress.

### DNA damage and defense

An extent of damage in an organism is manifestation of its capacity to cope with stress. According to reports in the literature, uridine phosphorylase2 (UPase2) plays critical roles in the metabolism of pyrimidines [56]. UPase2 regulates the balance of uridine concentration in plasma and tissues by catalyzing the dissimilation and phosphorylation of uridine to uracil for nucleic acid recovery and reuse [57]. Therefore, when UPase2 protein is up-regulated, it indicates that the catalytic reaction of uridine is strengthening, which leads to a decrease in uridine. Hence, in previous studies, when low-temperature stress was found to cause DNA damage in *T. fasciatus* [58], this might have been caused by an imbalance in uridine metabolism.

The immune and antioxidant systems also play pivotal roles in resisting potential damage caused by environmental stress. Vitamin intake is an important part of teleost immunity [59]. Retinol-binding protein (RBP) is a carrier of vitamin A that is mainly synthesized and released in liver [60]. RBP binds retinol and retinal, and as such is thought to play an important role in vitamin A (retinol) uptake, transport and metabolism [61]. Up-regulation of RBP enhances uptake of vitamin A to enhance fish immunity and growth [62]. For example, RBP was up-regulated in *Channa striatus* under high-temperature stress [63], suggesting that RBP has a positive effect on teleosts facing environmental temperature changes. In the vitamin digestion and absorption pathway, pantotherate (vitamin B5) and nicotinamide (vitamin B3) are up-regulated at low temperature, suggesting they might enhance low-temperature tolerance of *T. fasciatus* along with vitamin C [58]. Of course, the dosage of vitamins and their mechanism of action still need further study.

Carbohydrate metabolism is also a part of organism immunity. D-mannose is a hexose stimulating liver to secrete mannose-binding lectin (MBL), ultimately strengthening the immune system [64]. N-acetyl-D-glucosamine (GlcNAc) kinase is a hexokinase involved in carbohydrate metabolism. Considering our experimental results, D-mannose can be phosphorylated by GlcNAc kinase to form mannose-6-phosphate [65]. In mammals, mannose has a protective effect on an intestinal mucosal immune barrier in rats with severe acute pancreatitis [66]. On the other hand, acid phosphatase (ACP) is unique among lysosomal enzymes in that it has high concentration of both mannose and complex type sugars chains, and oligosaccharide chains of lysosomal enzymes contain a large proportion of mannose [67]. Therefore, the down-regulation of ACP decreases dephosphorylation of mannose [68]. Similarly, under low-temperature stress, ACP in sea bream (*Sparus aurata*) blood also decreased [69]. Interestingly, down-regulated ACP and GlcNAc kinase have opposite functions, namely dephosphorylation and phosphorylation, and the corresponding target metabolic substrate is up-regulated D-mannose. However, there is no evidence to support the competitive relationship between ACP and GlcNAc kinase. Obviously, the metabolic mechanism of D-mannose is still unclear under low-temperature stress, and more research is needed.

Oxidative stress is a physiological response of organisms to environmental changes. Detoxification-related protein glutathione S-transferase (GST) is an important indicator of the level of oxidative stress [70]. In our results, GST was up-regulated at both the mRNA and protein levels, indicating enhanced detoxification processes to remove ROS (reactive oxygen species). Correspondingly, the metabolism of glutathione disulfide was also enhanced as revealed by metabolomics. GST prevents damage to the cell membrane and other macromolecules by promoting the attachment of tripeptide GSH to a number of potentially harmful electrophilic substrates [71]. In *Danio rerio*, deficiency in the glucose transporter slc2al (Solute carrier family 2, facilitated glucose transporter member 1) has been shown to cause a suite of neural defects [72]. Therefore, up-regulation of sla2a1 in *T. fasciatus* may indicate a need to strengthen the nervous system under low-temperature stress. Not only that, Slc2a1 can also enhance the metabolism of L-leucine and acetylcarnitine in the cancer pathway and the insulin resistance pathway, respectively. However, this conclusion is derived from studies of human diseases and has not been confirmed in teleosts. A possibility needs to be explored that they may have a similar relationship in teleosts because we found that L-leucine could increase growth performance, improve metabolic activities and enhance non-specific immunities in tilapia [73] and black carp juveniles [74], and acetylcarnitine has antioxidant effects in teleosts [75].

## Conclusions

The results showed that transcriptome, proteome and metabolome of *T. fasciatus* liver changed significantly under low-temperature stress. Although more than 5000 genes were differentially expressed, only 55 relevant proteins (DEGs-DEPs) changed. Meanwhile, a total of 40 DEMs were found at the metabolic level. Through interaction analysis, we found that DEGs-DEPs and DEMs mainly regulate immunity, growth and antioxidant capacity by enhancing metabolism of unsaturated fatty acids, transport of bile salts, vitamin uptake, and antioxidant capacity, which ultimately affected cold tolerance of *T. fasciatus* (Fig. 4)*.* Co-expressed DEGs-DEPs in these biochemical processes can serve as potential indicators for regulation of cold-tolerant traits, and DEMs can be used as feed additives to investigate whether they can enhance cold tolerance of *T. fasciatus*. These findings provide new insights into the cold stress responses in fish and could promote research into the molecular breeding of pufferfish *Takifugu fasciatus*.

## Methods

### Experimental design and sampling

The sampling time point of stress was chosen basing on previous studies [29] and the results of our preliminary-experiment (Additional file 13: Table S11). The results indicated that the time point of 24 h under low temperature stress could be used as a breakthrough point in this study.

**Fig. 5:**
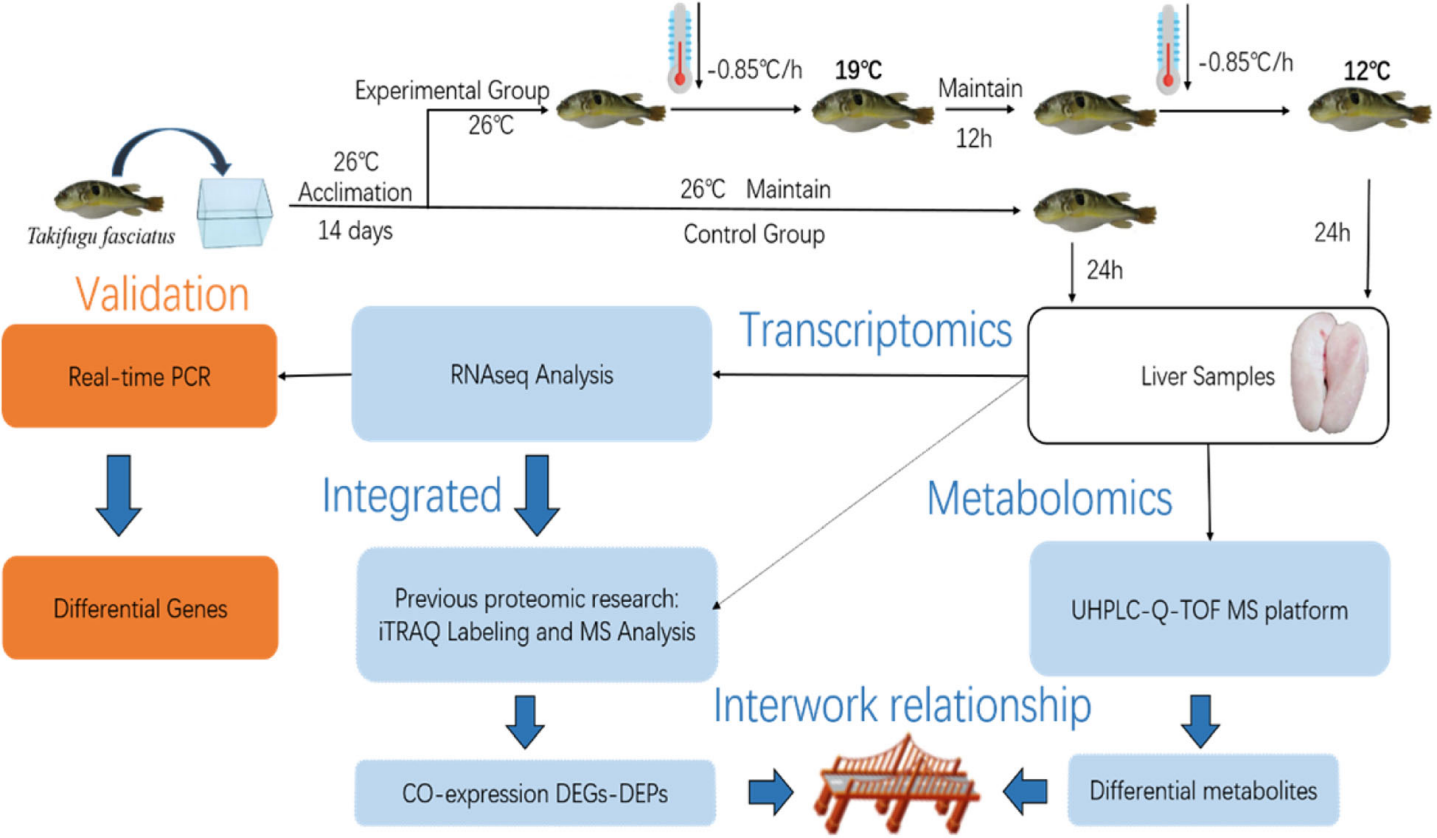
General workflow and summary of the present study

Taking experiment needs and the suitable culture density of juvenile *T. fasciatus* into consideration [28], a total of 120 juvenile *T. fasciatus* (13 ± 1.76 cm in length, 22 ± 2.85 g in weight), supplied from Zhongyang Group Co., Ltd. (Jiangsu Province, China), were randomly transferred to six aquaria with biofiltered water recirculation systems (equipped with cooling and heating functions; volume 200 L; flow rate 5 L/min), and were divided into the control group (G1, G2 and G3; total of 60 individuals; 2.2 ± 0.3 g/L) and the experimental group (Ga, Gb and Gc; total of 60 individuals; 2.2 ± 0.3 g/L). These individuals were acclimated at 26 ± 1 °C for 2 weeks. During the acclimation period, the commercial fish diet (including 42% w/w protein and 8.0% w/w fat, supplied by Zhenjiang Jiaji Feed Co. Ltd.) was given twice a day until 24 h before the experimental treatments (Fig. 5).

The water temperature of the CG was maintained at 26 ± 1 °C until the sampling was completed. The water temperature of the three experimental tanks (EG) decreased from 26 °C to 19 °C at a rate of 0.85 °C /1 h, and then maintained at 19 °C for 12 h, before decreasing from 19 °C to 12 °C at the same rate. After the EG was maintained at 12 °C for 24 h, both the experimental and control fish were euthanized with MS-222 at 10 mg L^− 1^, and quickly removed for liver dissection. Samples of EG (Ga, Gb and Gc) and CG (G1, G2 and G3) were done in three biological replicates. Each group (Ga, Gb, Gc, G1, G2 and G3) contains liver tissue from six different individuals, which were used for RNAseq and qPCR analysis. Ten replicates were consisted for both EG and CG respectively for metabolomics experiments. Ten individuals of EG came from Ga (3), Gb (3), and Gc (4). Similarly, 10 individuals from G1 (3), G2 (3) and G3 (4) were used as CG. Tissue samples were transferred to Eppendorf tubes, immediately flash frozen in liquid nitrogen, and then stored at − 80 °C.

### RNA isolation

Total RNA was extracted using Trizol reagent (Invitrogen, Carlsbad, CA, USA) following the manufacturer’s protocol. The total RNA quantity and purity were analysed with a Bioanalyzer 2100 and an RNA 6000 Nano LabChip Kit (Agilent, CA, USA) with RIN number > 7.0.

### Assembly and functional annotation of the transcriptome sequencing

For constructing six cDNA library, approximately 5 μg of total RNA per sample was used for the RNA sample preparations. The library for sequencing was generated using an Illumina TruSeq RNA Sample Preparation Kit (Illumina, San Diego, CA, USA). Transcriptome sequencing was carried out on an Illumina HiSeq 2500 platform that generated approximately 125-bp paired-end (PE) raw reads by LC Sciences (Houston, TX, USA). After removing adaptor sequences, ambiguous ‘N’ nucleotides (with the ratio of ‘N’ greater than 5%) and low quality sequences (with quality score less than 20), the high-quality trimmed sequences were used for further mapping to the *T. fasciatus* genome (unpublished data) with Hisat 2.0.4.

### Differential expression analysis

The expression level of each transcript was measured as the number of clean reads mapped to its sequence. The mapped clean read number was normalized to RPKM (reads per kilobase of transcript per million mapped reads) with RSEM 1.2.3. We used DESeq to determine the FDR threshold. FDR < 0.05 and fold change > 2 were considered to indicate significant expression abundance. All the DEGs identified in this study were used as reference for the enrichment analysis by GO (http://www.geneontology.org/) and KEGG (http://www.genome.jp/kegg/). Hypergeometric test was used to identify the overrepresented GO and KEGG pathway terms with a significance level at 0.05, and Beniamini & Hochberg method was used for the correction of the *p*-values.

### Extraction of metabolites

For metabolite extraction, 60 mg of each sample was weighted and placed on ice, then resuspended in 300 μL of 80% (vol/vol) methanol in water and homogenized by using an Ultra-Turrax homogenizer for 30s, with three 5-s pulses per sample, followed by ultrasonication at low temperature for 30 min, 2 times. Samples were kept at − 20 °C for 1 h and then spun at 4 °C for 15 min at 13, 000 rpm. Supernatant (250 μL per sample) was transferred to a new tube and dried down by using a speed vacuum at 30 °C. Dried samples were stored at − 80 °C.

### LC-MS/MS analysis (HILIC/MS)

Analyses were performed using an UHPLC (1290 Infinity LC, Agilent Technologies) coupled to a quadrupole time-of-flight (AB Sciex TripleTOF 6600).

For HILIC separation, samples were analyzed using a 2.1 mm × 100 mm ACQUIY UPLC BEH 1.7 μm column (Waters, Ireland). In ESI positive and negative modes, the mobile phase comprised A = 25 mM ammonium acetate and 25 mM ammonium hydroxide in water and B = acetonitrile. The gradients (vol/vol) were 85% B for 1 min, linearly reduced to 65% in 11 min, and then was reduced to 40% in 0.1 min and kept for 4 min, and then increased to 85% in 0.1 min, with a 5 min re-equilibration period employed.

The ESI source conditions were set as follows: Ion Source Gas1 (Gas1) as 60, Ion Source Gas2 (Gas2) as 60, curtain gas (CUR) as 30, source temperature: 600 °C, IonSpray Voltage Floating (ISVF) ±5500 V. In MS only acquisition, the instrument was set to acquire over the m/z range 60–1000 Da, and the accumulation time for TOF-MS scan was set at 0.20s/spectra. In auto MS/MS acquisition, the instrument was set to acquire over the m/z range 25–1000 Da, and the accumulation time for product ion scan was set at 0.05 s/spectra. The product ion scan was acquired using information dependent acquisition (IDA) with a high sensitivity mode selected. The collision energy (CE) was fixed at 35 V with ±15 eV. Declustering potential (DP) was set at ±60 V.

### Data processing

The raw MS data (wiff.scan files) were converted to MzXML files using ProteoWizard MSConvert and processed using XCMS for feature detection, retention time correction and alignment. The MS/MS spectra were searched in the in-house Standard MS/MS library. The library contained the MS/MS spectra of approximately 2700 compounds (primarily polar compounds and some nonpolar compounds), which were acquired using standards. The MS/MS spectra matching score was calculated using dot-product algorithm [76] taking fragments and intensities into account. The matching score cutoff was set as 0.8. The MS/MS spectra matching results were confirmed with standards. The data after completeness check, supplement missing value and delete extreme value were normalized between samples and metabolites respectively to ensure comparisons parallel. Finally, all extracted ion peak areas of the data were normalized and the data was subjected to Pareto-scaling processing in the SIMCA-P 14.1 software (Umetrics, Umea, Sweden).

In the extracted ion features, only the variables having more than 50% of the nonzero measurement values in at least one group were kept. For the multivariate statistical analysis, the MetaboAnalyst (www.metaboanalyst.ca) web-based system was used. After the Pareto-scaling, PCA and OPLS-DA were performed. The leave-one-out cross-validation and the response permutation testing were used to evaluate the robustness of the model. The significantly different metabolites were determined based on the combination of a statistically significant threshold of Variable Importance for the Projection (VIP) values obtained from the OPLS-DA model and two-tailed Student’s t test (*p* value) on the raw data. Metabolites with both multidimensional statistical analysis VIP > 1 and univariate statistical analysis *P* value < 0.05 were selected as metabolites with significant differences; VIP > 1 and 0.05 < *P* < 0.1 were used as differential metabolites (DEMs).

### Multi-omics analysis

Transcriptome samples used in this study are parallel to the materials researched in previous proteomic study [77]. Samples of EG and CG were done in three biological replicates, each containing liver tissue from six different individuals, and were used to conduct proteomic analysis by iTRAQ technique (Proteome data are available via ProteomeXchange with identifier PXD010955). A total of 160 proteins with fold change > 1.2 and a *p* -value < 0.05 were considered to be significantly differentially expressed (DEPs). These differential proteins were combined into the differential genes and metabolites obtained in this study to conduct the multi-omics analysis. First, the combined transcriptome and proteome were analyzed to find their co-expressed differential genes (proteins), and related pathways (related to DEGs-DEPs). Secondly, the analyses of proteomes and metabolomes were combined to identify pathways associated with them (related to DEPs-DEMs). Finally, the two result sets were combined to find regulatory pathways involving differentially expressed genes, proteins and metabolites. Moreover, the relationships among significantly different genes, proteins and metabolites were mapped into an interaction network by cytoscape software to provide rich and comprehensive information for revealing the molecular mechanisms of response to low-temperature stress at different levels.

### Validation of significant DEGs by RT-qPCR

A total of 11 differentially regulated mRNAs obtained from integrative analysis of multi-omics were verified with quantitative real-time PCR (qRT-PCR). Primers for qRT-PCR were listed in Additional file 14: Table S12. qRT-PCR with β-actin as an internal control was used to explore mRNA expression. qRT-PCR was performed with an SYBR Green Master kit according to the manufacturer’s protocol (Roche, Basel, Switzerland). The experiments were carried out in triplicate with a total volume of 20 μL in an ABI StepOnePlus, containing 10 μL SYBR Green Master, 4 μL cDNA (500 ng), and 3 μL forward and reverse primers (2 μmol/L). The qRT-PCR was programmed at 95 °C for 10 min, followed by 40 cycles of 95 °C for 15 s, and 55 °C for 1 min. The expression level was calculated by the 2^-△△CT^ method and subjected to statistical analysis.

### Detection and validation of SNPs in differential genes

By multi-omics analysis we obtained 14 differentially expressed genes, including 8 up-regulated and 3 down-regulated genes (Table 2). SNPs located on these genes have the most potential to be developed into molecular markers for breeding.

Potential SNPs were called using SAMtools software. SAMtools provides various utilities for manipulating alignments in the SAM format, including sorting, merging, indexing, and generating alignments in a preposition format. First, we called SNPs with the SAMtools mpileup utility. We then piped a BCF output file to SAMtools bcftools, which converted the BCF file into a VCF file. Afterwards, we piped the VCF file into vcfutils.pl with the varFilter-d100 option, which retained SNPs that had read depth higher than 100. In order to validate the accuracy of the SNPs predictions, fin tissue samples from 50 *T. fasciatus* were collected for this SNP validation. Genomic DNA was extracted from pterygiophore tissue samples, using a cell/tissue genomic DNA extraction kit (centrifugal column type, Generay PBiotech, Shanghai). Primers were designed to amplify the flanking sequence of the selected SNPs using Premier 5.0. The amplified PCR products were sequenced by utilizing an ABI3730 sequencer and the products were analyzed using DNAMAN 8.0.

## Additional files

Additional file 1: **Table S1.** Sequencing results of six cDNA libraries. (XLSX 10 kb)

Additional file 2: **Table S2.** Mapping results between transcriptome and reference genome. (XLSX 10 kb)

Additional file 3: **Table S3.** The results of transcriptome annotation referring to genome. (XLSX 10878 kb)

Additional file 4: **Figure S1.** Volcano plot of gene expression levels in EG (experimental group) compared with CG (control group). (PNG 122 kb)

Additional file 5: **Table S4.** GO analysis of differentially expressed genes. (XLSX 191 kb)

Additional file 6: **Figure S2.** GO analysis of significant differentially expressed genes. (PNG 212 kb)

Additional file 7: **Table S5.** KEGG analysis of differentially expressed genes. (XLSX 16 kb)

Additional file 8: **Table S6.** The classification of integrated analysis results on transcriptome and proteome. (XLSX 308 kb)

Additional file 9: **Table S7.** qPCR verification results of transcriptomes. (DOCX 15 kb)

Additional file 10: **Table S8.** The list of SNPs. (XLSX 19240 kb)

Additional file 11: **Table S9.** Location information of the predicted SNPs. (DOCX 15 kb)

Additional file 12: **Table S10.** Primers for detecting SNPs. (DOCX 15 kb)

Additional file 13: **Table S11.** The results of preliminary experiment. (DOCX 15 kb)

Additional file 14: **Table S12.** Primers for verifying the transcriptome. (DOCX 15 kb)

Additional file 15: **Table S13.** The metabolite data set of this study. (XLSX 3761 kb)

## Data Availability

The dataset generated by deep-sequencing platforms was deposited in the NCBI Sequence Read Archive (SRA, http://www.ncbi.nlm.nih.gov/Traces/sra). The NCBI Sequence Read Archive accession number is GSE129226. The mass spectrometry proteomics data supporting the conclusions of this article is available in ProteomeXchange via the PRIDE (http://www.ebi.ac.uk/pride) partner repository with the dataset identifier PXD010955. The metabolite data set supporting the results of this article is included within the article, and can be found in the Supplemental Information (Additional file 15: Table S13).

## Abbreviations

AACS: Acetoacetyl-CoA synthetase
ACAD: Acyl-CoA desaturase
ACP: Acid phosphatase
ARA: Arachidonic acid
ATP5J: ATP synthase-coupling factor 6
BSEP: Bile salt export pump
cMOAT: Canalicular multispecific organic anion transporter
DEGs: Differentially expressed genes
DEGs-DEPs: Differentially co-expressed genes and proteins
DEMs: Differential metabolites
DEPs: Differentially expressed proteins
DEPs_DEGs_Opposite: Differentially expressed proteins and genes with opposite trends; DEPs_NDEGs Differential expressedly proteins with no significant difference in transcription level
DEPs_DEGs_SameTrend: differentially expressed proteins and genes with the same trend
DEPs-DEMs: Associated differential proteins and differential metabolites
DHA: (4Z,7Z,10Z,13Z,16Z,19Z)-4,7,10,13,16,19-docosahexaenoic acid
G proteins: Guanine nucleotide-binding proteins subunit base 4
GlcNAc: N-acetyl-D-glucosamine
GO: Gene Ontology
GST: Glutathione S-transferase
HSL: Hormone-sensitive lipase
KEGG: Kyoto Encyclopedia of Genes and Genomes
NDEPs_DEGs: Non differentially expressed proteins with significant difference in transcription level
NDEPs_NDEGs: Genes with no significant difference in transcription and protein levels
RBP: retinol-binding protein
ROS: Reactive oxygen species
slc2a1: Solute carrier family 2, facilitated glucose transporter member 1
SNP: Single nucleotide polymorphisms
Ubap1: Ubiquitin-associated protein 1-like
UPase: Uridine phosphorylase
VIP: Variable Importance for the Projection
