# Supplementary figures and images for: Integrated application of multi-omics provides insights into cold stress responses in pufferfish Takifugu fasciatus
Source: BMC Genomics. 2019 Jul 8;20:563. doi: 10.1186/s12864-019-5915-7 (PMC6615287; doi:10.1186/s12864-019-5915-7)

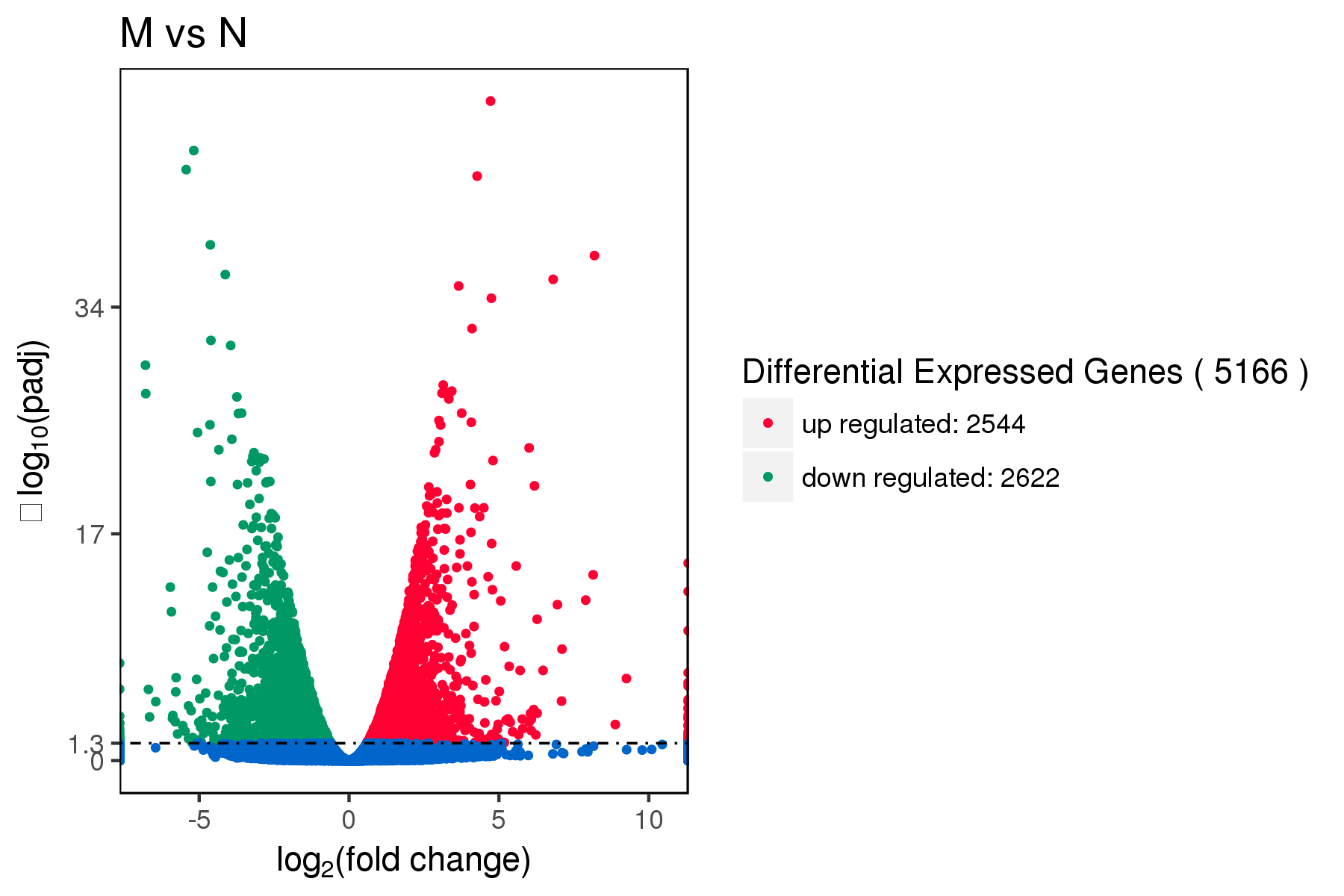

Supplement: Supplementary file 4 — Figure S1. Volcano plot of gene expression levels in EG (experimental group) compared with CG (control group). (PNG 122 kb) [file 12864_2019_5915_MOESM4_ESM.png]

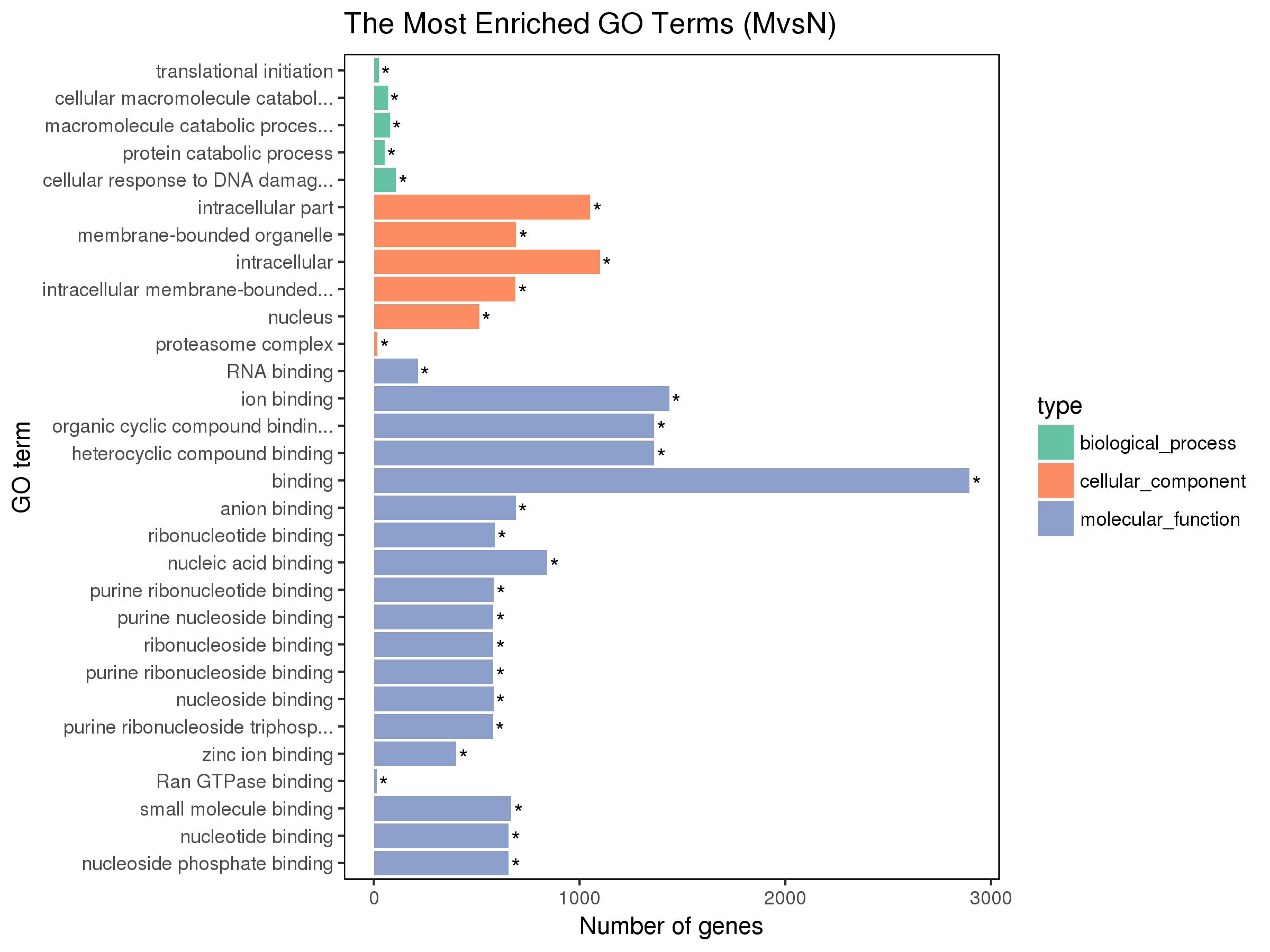

Supplement: Supplementary file 6 — Figure S2. GO analysis of significant differentially expressed genes. (PNG 212 kb) [file 12864_2019_5915_MOESM6_ESM.png]
