# Supplementary material for: Integrated application of multi-omics provides insights into cold stress responses in pufferfish Takifugu fasciatus
Source: BMC Genomics. 2019 Jul 8;20:563. doi: 10.1186/s12864-019-5915-7 (PMC6615287; doi:10.1186/s12864-019-5915-7)
Supplement: Supplementary file 9 — Table S7. qPCR verification results of transcriptomes. (DOCX 15 kb) [file 12864_2019_5915_MOESM9_ESM.docx]

Table S7 qPCR verification results of transcriptomes

| Gene abbreviation | Transcriptomic (log2foldchange) | Regulation | qPCR (log2fold change) |
| --- | --- | --- | --- |
| BESP | 1.12 | Up | 1.21* |
| RBP | 1.75 | Up | 2.02* |
| GST | 1.30 | Up | 1.42* |
| UPase | 1.99 | Up | 2.07* |
| slc2a1 | 1.82 | Up | 2.03* |
| Ubapl | 1.73 | Up | 1.84* |
| ACAD | 2.88 | Up | 2.61* |
| ATP5J | 1.97 | Up | 2.07* |
| ACP | -1.90 | down | -1.76* |
| G proteins | -1.20 | down | -1.27* |
| GlcNAc | -1.91 | down | -1.97* |

The asterisks represent statistically significant differences between EG versus CG (p < 0.05).
