# Supplementary material for: Integrated application of multi-omics provides insights into cold stress responses in pufferfish Takifugu fasciatus
Source: BMC Genomics. 2019 Jul 8;20:563. doi: 10.1186/s12864-019-5915-7 (PMC6615287; doi:10.1186/s12864-019-5915-7)
Supplement: Supplementary file 11 — Table S9. Location information of the predicted SNPs. (DOCX 15 kb) [file 12864_2019_5915_MOESM11_ESM.docx]

Table S11 Location information of the predicted SNP

| Gene | Regulated | SNP | | POS | chromosome |
| --- | --- | --- | --- | --- | --- |
|  |  | REF | ALT |  |  |
| Solute carrier family 2, facilitated glucose transporter member 1 | Up | C | T | 1314601 | Scaffold64 |
|  |  | A | T | 1315433 | Scaffold64 |
|  |  | T | C | 1316005 | Scaffold64 |
|  |  | T | C | 1316524 | Scaffold64 |
|  |  | T | C | 1316527 | Scaffold64 |
|  |  | G | A | 1316807 | Scaffold64 |
|  |  | C | A | 1316913 | Scaffold64 |
| Glutathione S-transferase omega-1 | Up | G | A | 401415 | Scaffold6 |
|  |  | A | G | 401487 | Scaffold6 |
|  |  | C | G | 401502 | Scaffold6 |
|  |  | T | C | 402432 | Scaffold6 |
| Bile salt export pump | Up | T | C | 4150355 | Scaffold30 |
|  |  | T | C | 4150487 | Scaffold30 |
|  |  | C | G | 4150520 | Scaffold30 |
| N-acetyl-D-glucosamine kinase | Down | G | C | 1108787 | Scaffold49 |
|  |  | A | C | 1108971 | Scaffold49 |
|  |  | C | T | 1109151 | Scaffold49 |
|  |  | A | G | 1109366 | Scaffold49 |
|  |  | A | T | 1109390 | Scaffold49 |
|  |  | T | C | 1109550 | Scaffold49 |
|  |  | C | T | 1109721 | Scaffold49 |
|  |  | A | G | 1110069 | Scaffold49 |
| Guanine nucleotide-binding protein subunit beta-4 | Down | G | A | 273770 | Scaffold29 |
| Uridine phosphorylase 2 | Up | None | | - | - |
| Retinol-binding protein 2 | Up | None | | - | - |
| Ubiquitin-associated protein 1-like | Up | None | | - | - |
| ATP synthase-coupling factor 6 | Up | None | | - | - |
| Acyl-CoA desaturase | Up | None | | - | - |
| Acid phosphatase | Down | None | | - | - |
