# Supplementary material for: Integrated application of multi-omics provides insights into cold stress responses in pufferfish Takifugu fasciatus
Source: BMC Genomics. 2019 Jul 8;20:563. doi: 10.1186/s12864-019-5915-7 (PMC6615287; doi:10.1186/s12864-019-5915-7)
Supplement: Supplementary file 12 — Table S10. Primers for detecting SNPs. (DOCX 15 kb) [file 12864_2019_5915_MOESM12_ESM.docx]

Table S10 Sequence of primer for detecting SNPs

| Gene | Pos | Primer sequences (5′-3′) |
| --- | --- | --- |
| slc2a1 | 1314601 | F: CGCATTCTCGCCTTCTGTC  R: TACGCCTGGGGAGGTGAG |
|  | 1315433 | F: AGGAAGCCAGGAAAGGTCG  R: AGCGTTGATGCCCGACAG |
|  | 1316005 | F: AGCCTGTAGACCTGAGGAGCA  R: CTGATAACCCAGGATAGGAGACAT |
|  | 1316524-7 | F: TTGAGCAGAAGAGCAACCAGTC  R: ATATGGGCCGCAAAGTTCC |
|  | 1316807 | F: GAACTTTGCGGCCCATATG  R: ACATGGATGCTCAGTGCTTTTC |
|  | 1316913 | F: GTGCCCGAGACCAAAGGAA  R: ATTTTCCTCTCTCGTAATCTCAACTT |
| GST | 401415 | F: TTATTGGATGCTGAAAATCTAGTTACA  R: CTGGCAAAGTGGACTGAACG |
|  | 401487-502 | F: GATAGAAAAGACCAAGATGACAGAAAC  R: AATTGTTTCAGAGCATATTGGTTTT |
|  | 402432 | F: GGAAGCAGTTTCTTTTCAGGGTA  R: GGTGTTGAATGCCAAAGGG |
| BSEP | 4150355 | F: CCAACCAATCACGGCGAC  R: GGTCCTGTGGGTACGTGGG |
|  | 4150487-520 | F: TGCTCCAATTCCGACGTAATAG  R: TGCTCACGGACACCTTCATC |
| GlcNAc | 1108787 | F: CCCACCCAAACTTTCCAGC  R: GTTGGGAACTGCTCCTTCATC |
|  | 1108971 | F: AGGACTGGACCCCAACACTC  R: CCTGGAATAAAGGTTGGGACA |
|  | 1109151 | F: ATCGGTGCAATGGCAACG  R: ATACATGCTGTTGCTATGGTTACCT |
|  | 1109366-90 | F: CATGATGGGTGATGAAGGCTC  R: GTAGAGGAGCGGTAGAATGCC |
|  | 1109550 | F: CCTGGTAGCGCCTCCTCAT  R: GAATGAAGAAGTGCTGGCAGAG |
|  | 1109721 | F: GGGCATTCTACCGCTCCTC  R: AGGGTGCCTCAACAGAGCC |
|  | 1110069 | F: GGGAGCTTCTGAAACCTGGTC  R: CATACTGATGGTGGCACCTATATTC |
| G protein | 273770 | F: GCATTCATCTAAAATCTATTCTTCGT  R: AAAGTCAGAACAATGAAAATCCGT |
