# Supplementary material for: Integrated application of multi-omics provides insights into cold stress responses in pufferfish Takifugu fasciatus
Source: BMC Genomics. 2019 Jul 8;20:563. doi: 10.1186/s12864-019-5915-7 (PMC6615287; doi:10.1186/s12864-019-5915-7)
Supplement: Supplementary file 13 — Table S11. The results of preliminary experiment. (DOCX 15 kb) [file 12864_2019_5915_MOESM13_ESM.docx]

In the experimental group (EG, 12℃± 1℃) and control group (CG, 26℃± 1℃), fish livers were collected to assay ROS (reactive oxy gen species), SOD (superoxide dismutase), GSH (glutathione) and GSH-PX (glutathione peroxidase) by the test kits (Jiancheng Bioengineering, Nanjing, China) at 0 h, 6 h, 24 h and 7 d. The results showed that the oxidative stress was most severe after 24 h exposure to low temperature (Additional file 13: Table S11). Also, when temperature was maintained at 12 ℃ for 24 h, the swimming frequency of *T. fasciatus* decreased significantly. Therefore, the time point of 24 h was used as a breakthrough point in this study.

Table S11 The fold change of ROS, SOD, GSH-PX and GSH at different time points

|  | ROS | SOD | GSH-PX | GSH |
| --- | --- | --- | --- | --- |
| 0h | 1.74 ^b^ | 1.56 ^b^ | 1.41 ^b^ | 1.81^b^ |
| 6h | 1.78 ^b^ | 0.74 ^d^ | 1.07 ^d^ | 2.04^a^ |
| 24h | 2.01 ^a^ | 1.73 ^a^ | 1.63 ^a^ | 2.31^a^ |
| 7d | 1.52 ^c^ | 1.49 ^c^ | 1.61^a^ | 1.43^c^ |

The ratio of enzyme activity of each population in the treatment (EG, 12℃) and the control (CG, 26℃) at the corresponding time points was used to reflect the treatment effects on antioxidative enzymes in the *T. fasciatus* liver under low temperature stress. Data shown are means of at least three independent experiments. Significant differences (P < 0.05) among the four time points were indicated by different letters
