# Supplementary material for: Integrated application of multi-omics provides insights into cold stress responses in pufferfish Takifugu fasciatus
Source: BMC Genomics. 2019 Jul 8;20:563. doi: 10.1186/s12864-019-5915-7 (PMC6615287; doi:10.1186/s12864-019-5915-7)
Supplement: Supplementary file 14 — Table S12. Primers for verifying the transcriptome. (DOCX 15 kb) [file 12864_2019_5915_MOESM14_ESM.docx]

Table S9 Sequence of primer for verifying the transcriptome

| Name | Primer sequences (5′-3′) | Application |
| --- | --- | --- |
| BESP | F: ACGCCAAAGCCAAGATCTCA  R: GTGTGATCGCACATTACCGC | qRT-PCR. |
| RBP | F: GAAAGCCCTCGACATCGACT  R: GCGTCATGACCTTTCGGTTG | qRT-PCR. |
| GST | F: ACTACATGATGTGGCCGTGG  R: TACGAGGTAGCTTTGACCGC | qRT-PCR. |
| UPase | F: CTTCCCCACCGTGATTGGAA  R: CACTCCAGCCTCGTATGCTT | qRT-PCR. |
| slc2a1 | F: ACAGGTCTTCGGTCTGGAGT  R: TTTGAGCACGATCAGCAGGT | qRT-PCR. |
| Ubapl | F: ATGGTGAGGAGCTTCAGTGC  R: TCGGACGTGTTTCCATCGTT | qRT-PCR. |
| ACAD | F: GTCGACGGTGGAGGATGTTT  R: AGCAAACGGCACTCCATACA | qRT-PCR. |
| ATP5J | F: GCTCGACCCTGTCCAGAAAT  R: ACTTGGTGAAGTCTCCCCCT | qRT-PCR. |
| ACP | F: TGAAGTCAGTCTGGAGCGTG  R: CGGAGCCATTGTCGTTTGTG | qRT-PCR. |
| G proteins | F: TATGCAATGCACTGGGGGAG  R: TAATTCCCAGAGGGGGCGTA | qRT-PCR. |
| GlcNAc | F: AAGCTGGTTAACCCAGACGG  R: TAGGCCTCCATGGCTTTTCG | qRT-PCR. |
